# ^13^C metabolic flux analysis shows that resistin impairs the metabolic response to insulin in L6E9 myotubes

**DOI:** 10.1186/s12918-014-0109-z

**Published:** 2014-09-14

**Authors:** Shirley Guzmán, Silvia Marin, Anibal Miranda, Vitaly A Selivanov, Josep J Centelles, Romain Harmancey, Fatima Smih, Annie Turkieh, Yves Durocher, Antonio Zorzano, Philippe Rouet, Marta Cascante

**Affiliations:** 1 Department of Biochemistry and Molecular Biology, Faculty of Biology, Universitat de Barcelona, Av Diagonal 643, Barcelona, 08028, Spain; 2 Institute of Biomedicine of Universitat de Barcelona (IBUB) and CSIC-Associated Unit, Barcelona, Spain; 3 Institut National de la Santé et de la Recherche Médicale (INSERM), UMR 1048, Toulouse, France; 4 Université Toulouse III Paul-Sabatier, Institut des Maladies Métaboliques et Cardiovasculaires (I2MC), Equipe n°7, Toulouse, France; 5 Animal Cell Technology Group, Biotechnology Research Institute, National Research Council Canada, Montreal, QC, Canada; 6 Institute for Research in Biomedicine (IRB Barcelona) and CIBER of Diabetes and Associated Metabolic Diseases (CIBERDEM), Barcelona, Spain

**Keywords:** Resistin, Insulin resistance, Glucose metabolism, Fluxomics, Bioinformatics, Tracer-based metabolomics

## Abstract

**Background:**

It has been suggested that the adipokine resistin links obesity and insulin resistance, although how resistin acts on muscle metabolism is controversial. We aimed to quantitatively analyse the effects of resistin on the glucose metabolic flux profile and on insulin response in L6E9 myotubes at the metabolic level using a tracer-based metabolomic approach and our *in-house* developed software, Isodyn.

**Results:**

Resistin significantly increased glucose uptake and glycolysis, altering pyruvate utilisation by the cell. In the presence of resistin, insulin only slightly increased glucose uptake and glycolysis, and did not alter the flux profile around pyruvate induced by resistin. Resistin prevented the increase in gene expression in pyruvate dehydrogenase-E1 and the sharp decrease in gene expression in cytosolic phosphoenolpyruvate carboxykinase-1 induced by insulin.

**Conclusions:**

These data suggest that resistin impairs the metabolic activation of insulin. This impairment cannot be explained by the activity of a single enzyme, but instead due to reorganisation of the whole metabolic flux distribution.

## 1 Background

Obesity is increasing to epidemic proportions [1],[2], starting from young ages [3],[4], and is associated with an increase in the prevalence of type-2 diabetes mellitus (T2DM) throughout the world [2],[5]. In recent years, active participation of immune cells in obesity and T2DM has become evident [6],[7]. This chronic activation of the innate immune system can lead to insulin resistance (IR), impaired tolerance to glucose and, eventually, T2DM [8],[9].

Adipose tissue produces a vast array of adipocyte-derived factors (“adipokines”) that regulate the metabolism, inflammation and body mass [10],[11]. One of these adipokines, resistin, belongs to a family of cysteine-rich proteins shown to be involved in inflammation and altered insulin sensitivity in rodents [12],[13]. Resistin is produced from adipose tissue in rodents whereas, in humans, it is secreted by the mononuclear lymphocytes and stromal cells within adipose tissue [14],[15]. Studies in humanised resistin mice suggest that, even though the site of resistin production differs between species, human resistin exacerbates inflammation in white adipose tissue and contributes to IR, thereby impairing its normal effects [16],[17].

IR is defined as the genetic or learned inability of target tissues to respond normally to the action of circulating hormones. It has been described in skeletal muscle but also in liver and adipose tissues [18]. Some studies have shown that resistin affects glucose transport and insulin-stimulated oxidation of glucose in L6 skeletal muscle cells [19]-[22]. It also decreases the uptake and oxidation of long-chain fatty acids [23] and glycogen synthase kinase-3-β, as well as insulin-stimulated insulin receptor substrate-1 (IRS-1) tyrosine phosphorylation [19],[22] in the same cell line. Furthermore, high levels of resistin in rats leads to IR involving impaired insulin signalling in skeletal muscle, liver and adipose tissues, resulting in glucose intolerance, hyperinsulinemia and hypertriglyceridemia [24]. These observations link resistin in muscle-cell metabolism to IR.

Changes in the metabolome (i.e., the whole set of metabolites) have been described as the “ultimate” response of an organism to various events, such as genetic alterations as well as disease-based or environmental influences [25]. However, the metabolome is dynamic and metabolites are transformed continuously in the cell. Comprehensive characterisation of the metabolic networks and their functional states requires quantitative knowledge of intracellular metabolic fluxes. These intracellular fluxes can be quantified by analysing incorporation of labelled substrates in metabolic products using appropriate bioinformatic tools [26].

Skeletal muscle is considered to be the main tissue involved in the maintenance of glucose homeostasis because its contribution to glucose uptake is ≈ 75% of the total contribution of peripheral tissues [27], and it is the main tissue responsible for insulin-dependent glucose use. Also, pyruvate homeostasis-related fluxes in skeletal muscle play a critical part in glucose homeostasis [28]-[32]. A better understanding of how these fluxes work together in muscle cells is needed to understand this process.

The aim of the present study was to examine the effect of resistin on the central carbon metabolic network of rat skeletal muscle cells, and to ascertain how resistin alters the response of muscle cells to insulin at the metabolic level. We used [1,2-^13^C_2_]-glucose as a tracer, analysed isotopomer distributions by gas cromatography (GC) coupled with mass spectrometry (MS), and quantified metabolic fluxes using Isodyn software [26],[33]-[36]. In the model of the glucose metabolic network in skeletal muscle we included the glycolytic pathway and pentose phosphate pathway (PPP). Pyruvate dehydrogenase complex (PDC)-, phosphoenolpyruvate carboxykinase (PEPCK)- and pyruvate carboxylase (PC)-catalysed reactions were also included to account for pyruvate homeostasis-related fluxes. Resistin-induced alterations in the profile of metabolic fluxes of rat skeletal muscle cells were identified, and were complemented by analyses of the expression of certain genes.

The study presented here offers a wider vision than previously published of the metabolic reorganisation that resistin induces in rat skeletal muscle cells and in the metabolic response of these cells to insulin. Our observations provide new data for a more precise understanding of the effect of resistin on metabolic-network adaptations of skeletal muscle cells, and help to explain the role of resistin in the IR described in myocytes. Our work reveals the importance of tackling the study of complex biological systems from a systems-biology point of view to fully understand multifactorial diseases and identify new therapeutic targets.

## 2 Results

For analyses of the effect of resistin on glucose metabolism and on the metabolic response of muscle cells to insulin, L6E9 myotubes were pretreated for 8 h with or without resistin (100 nM). After preincubation, myotubes were incubated for a further 6 h with glucose (10 mM) that was 50% enriched in [1,2-^13^C_2_]-glucose in the absence or presence of 100 nM insulin and in the continued presence or absence of resistin.

**Figure 1:**
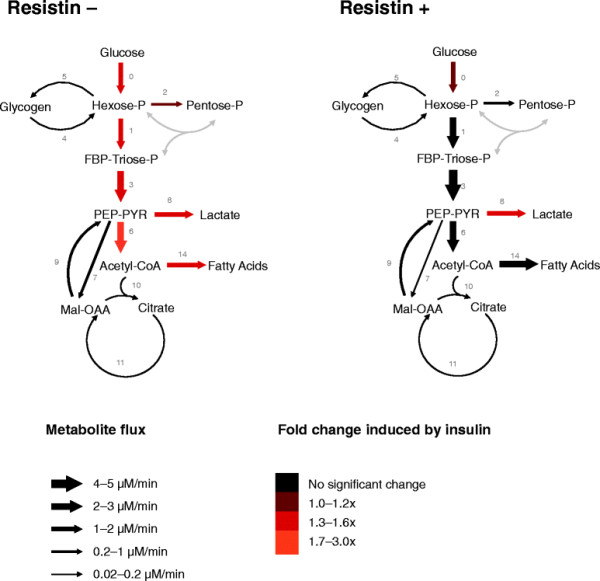
**Metabolic fluxes in L6E9 myotubes not treated and treated with resistin and/or insulin.** L6E9 myotubes were treated (resistin+) or not treated (resistin–) with 100 nM resistin for 8 h and then incubated for 6 h with 10 mM glucose, 50%-enriched in [1,2-^13^C_2_]-D-glucose in the absence or in the presence of 100 nM insulin. Fluxes were estimated using the software Isodyn. Arrow sizes indicate net fluxes in L6E9 myotubes not treated or treated with resistin that were incubated in the absence of insulin. Colours indicate flux fold-changes in response to insulin. Fluxes plotted are the median values from the 20 best flux sets (Table 3). Grey-coloured fluxes have not been measured. FBP, fructose-1,6-bisphosphate; Hexose-P, glucose-6-phosphate and its isomers; Mal, malate; OAA, oxaloacetate; Pentose-P, ribose-5-phosphate and its isomers; PEP, phosphoenolpyruvate; PYR, pyruvate; Triose-P, glyceraldehyde-3-phosphate and dihydroxyacetone phosphate.

After 6 h of incubation, glucose consumption, lactate production, intracellular levels of glycogen and glucose-6-phosphate (G6P) were determined (Table 1), as were the mass-isotopomer distributions in glucose and lactate from the incubation medium, as well as glycogen glucose and ribose isolated from RNA in cell pellets (Table 2). Analyses of the obtained data by Isodyn determined the distribution of the metabolic flux profile of L6E9 myotubes under various incubation conditions (Table 3). Figure 1 summarises the qualitative changes in metabolites and fluxes.

**Table 1:** Biochemical parameters in L6E9 myotubes after different incubations with resistin and/or insulin

	**Incubation condition**			
	**Ins-**		**Ins+**	
	**Res-**	**Res+**	**Res-**	**Res+**
**Glucose consumption (mM)**	0.50 ± 0.04	0.61 ± 0.06*	0.78 ± 0.03**	0.70 ± 0.09
**Lactate production (mM)**	0.48 ± 0.01	0.56 ± 0.02**	0.75 ± 0.06**	0.79 ± 0.02^†^
**Glycogen (μg glucose/mg prot)**	9.88 ± 1.99	9.97 ± 0.98	11.34 ± 0.59	11.62 ± 1.21
**Glucose-6-phosphate (nmol/mg prot)**	0.92 ± 0.14	1.44 ± 0.28	2.30 ± 0.29**	2.10 ± 0.19^†^

L6E9 myotubes were treated (Res+) or not treated (Res–) with 100 nM resistin for 8 h and then incubated for 6 h in the absence (Ins–) or presence (Ins+) of 100 nM insulin. Glucose consumption, lactate production, and intracellular contents of glycogen and glucose-6-phosphate were determined as described in the Methods section. Results are the mean ± standard deviation (n = 3). Units of glucose consumption and lactate production refer to the volume of the incubation medium. (*) indicates p < 0.05 and (**) p < 0.01 compared with the condition of neither resistin nor insulin. (†) indicates p < 0.05 compared with the L6E9 myotubes treated only with resistin.

**Table 2:** Mass isotopomer distribution of lactate, glycogen and ribose after incubations with resistin and/or insulin

	**Mass isotopomer distributions (%)**			
	**Incubation condition**			
	**Ins-**		**Ins+**	
	**Res-**	**Res+**	**Res-**	**Res+**
***Lactate***				
**m0**	88.56 ± 0.98	88.74 ± 0.25	87.68 ± 0.22	87.63 ± 0.17
**m1**	0.70 ± 0.34	0.48 ± 0.11	0.63 ± 0.06	0.52 ± 0.14
**m2**	10.53 ± 0.30	10.85 ± 0.15	11.69 ± 0.05*	11.78 ± 0.11
**m3**	0.21 ± 0.63	0.07 ± 0.18	0.00 ± 0.11	0.07 ± 0.13
***Glycogen***				
**m0**	67.28 ± 0.65	69.20 ± 1.41	65.14 ± 1.08	64.48 ± 0.45
**m1**	0.29 ± 0.03	0.36 ± 0.14	0.96 ± 0.10	0.84 ± 0.10
**m2**	31.78 ± 0.74	29.91 ± 1.02	33.20 ± 1.18	33.78 ± 0.76
**m3**	0.53 ± 0.19	0.43 ± 0.28	0.43 ± 0.07	0.60 ± 0.20
**m4**	0.00 ± 0.02	0.00 ± 0.06	0.17 ± 0.05	0.15 ± 0.02
***Ribose***				
**m0**	96.13 ± 0.55	94.28 ± 0.76	94.47 ± 0.48	92.83 ± 1.18
**m1**	1.34 ± 0.23	1.45 ± 0.11	2.56 ± 0.14	2.97 ± 0.15
**m2**	0.68 ± 0.19	1.14 ± 0.16*	1.18 ± 0.09*	1.40 ± 0.23
**m3**	1.17 ± 0.63	1.56 ± 0.58	0.87 ± 0.17	1.14 ± 0.32
**m4**	0.50 ± 0.44	1.17 ± 0.22*	0.61 ± 0.14	1.17 ± 0.41

L6E9 myotubes were treated (Res+) or not treated (Res–) with 100 nM resistin for 8 h and then incubated for 6 h with 10 mM glucose, 50%-enriched in [1,2-^13^C_2_]-glucose in the absence (Ins–) or presence (Ins+) of 100 nM insulin. Mass isotopomer distributions at the end of incubations were determined, as described in the Methods section. Results are the mean ± standard deviation (n = 3). (*) indicates p < 0.05 compared with the condition of neither resistin nor insulin.

**Table 3:** Metabolic fluxes adjusted by Isodyn for different incubation conditions

		**Metabolic fluxes (nmol · mL**^**-1**^ **· min**^**-1**^**)**							
		**Incubation condition**							
		**Ins –**				**Ins +**			
**Flux num**	**Flux reaction**	**Res –**		**Res +**		**Res –**		**Res +**	
		**Median**	**[min–max]**	**Median**	**[min–max]**	**Median**	**[min–max]**	**Median**	**[min–max]**
0	Glucose phosphorylation	1.500	[1.500–1.500]	1.700**	[1.700–1.700]	2.300**	[2.300–2.300]	1.975^††^	[1.975–1.975]
***Glycolytic and PPP fluxes*:**									
1	Phosphofructokinase	1.674	[1.442–3.004]	2.018	[1.679–2.586]	2.664*	[2.537–2.670]	1.907	[1.874–1.919]
2	Oxidative branch of PPP	0.199	[0.196–0.199]	0.200	[0.198–0.200]	0.248**	[0.246–0.298]	0.198	[0.192–0.199]
3	Pyruvate kinase	3.078	[3.026–3.262]	3.582	[2.644–4.126]	4.671**	[4.573–4.873]	4.337	[4.087–4.468]
***Glycogen-related fluxes*:**									
4	Glycogen phosphorylase	0.038	[0.034–0.052]	0.034	[0.024–0.066]	0.043	[0.037–0.046]	0.032	[0.029–0.035]
5	Glycogen synthase	0.040	[0.036–0.053]	0.035	[0.024–0.067]	0.050	[0.043–0.058]	0.038	[0.034–0.040]
***Pyruvate homeostasis-related fluxes*:**									
6	Pyruvate dehydrogenase complex	1.511	[1.421–1.843]	2.354**	[2.118–2.363]	2.479*	[1.785–2.987]	2.125	[1.575–2.494]
7	Pyruvate carboxylase	0.200	[0.113–0.291]	0.117	[0.027–0.201]	0.197	[0.155–0.254]	0.031	[0.028–0.055]
8	Lactate dehydrogenase	1.366	[1.292–1.451]	1.511	[1.446–1.654]	2.033**	[1.620–2.738]	2.192^††^	[1.722–2.739]
9	Pyruvate cycling	0.228	[0.200–0.377]	0.474	[0.222–0.884]	0.296	[0.264–0.389]	0.538	[0.303–0.594]
***Tricarboxylic acid cycle fluxes*:**									
10	Citrate synthase	0.022	[0.019–0.386]	0.012	[0.001–0.023]	0.099	[0.022–0.172]	0.009	[0.002–0.037]
11	Citrate - > Malate	0.035	[0.032–0.462]	0.072	[0.050–0.400]	0.185	[0.110–0.298]	0.370	[0.082–0.395]
12	Malate - > OAA	0.640	[0.481–2.088]	1.152	[0.453–3.104]	1.509	[0.780–2.413]	2.078	[0.477–3.095]
13	OAA - > Malate	0.605	[0.446–1.776]	1.080	[0.294–2.800]	1.324	[0.668–2.115]	1.708	[0.336–2.773]
14	Acetyl-CoA output	1.489	[1.313–1.625]	2.344**	[2.114–2.355]	2.380**	[1.760–2.851]	2.116	[1.565–2.485]

Principal fluxes of central carbon metabolism calculated in L6E9 myotubes treated (+Res) or not treated (–Res) with 100 nM resistin for 8 h and then incubated for 6 h with 10 mM glucose, 50%-enriched in [1,2-^13^C_2_]-glucose in the absence (–Ins) or presence (+Ins) of 100 nM insulin. Glucose phosphorylation flux was fixed according to the glucose consumption measured (Table 1). Remaining fluxes are expressed by median values as well as minimum and maximum values from the 20 best flux sets. Fluxes are expressed in nmol · mL^–1^ · min^–1^, where volume is the volume of the incubation medium. χ^2^ values for the averages were: Ins– Res–: 11.35; Ins– Res+: 5.42; Ins + Res–: 22.50; Ins + Res+: 25.56. (**) indicates that the flux is different to the control condition with a 99% confidence interval (CI), (*) indicates that the flux is different to the control condition with a 95% CI, and (††) indicates that the flux differs from the condition in which cells were treated only with resistin with a 99% CI. PPP, pentose phosphate pathway; OAA, oxaloacetate.

### 2.1 Basal metabolic flux profile of L6E9

Isodyn quantified the metabolic-flux distribution in L6E9 myotubes incubated in the presence of glucose (10 mM) and in the absence of insulin or resistin (Table 3, first column). Under this incubation condition, cells had a glucose phosphorylation of 1.500 nmol · mL^–1^ · min^–1^. This flux was maintained throughout the subsequent steps of “upper glycolysis” (of which flux-1 is representative) until the split of fructose-1,6-bisphosphate into two triose-phosphates by aldolase. From this reaction onwards, the glycolytic flux (hereafter referred to as “lower glycolysis”) increased twofold (as shown in flux-3). Additionally, the flux across the oxidative branch of the PPP was around eight-times lower than the upper glycolytic flux. With regard to glycogen metabolism, glycogen phosphorylase (GP) (flux-4) and glycogen synthase (GS) (flux-5) were active, and glycogen recycling was estimated to be ≈ 2% of the total glucose phosphorylation flux.

However, the most important contribution of Isodyn for interpretation of experimental data was the possibility of flux quantification around the final steps of lower glycolysis and the tricarboxylic acid (TCA) cycle. In that regard, Isodyn revealed that ≈ 49% of phosphoenolpyruvate (PEP) and pyruvate entered the TCA cycle through the reactions catalysed by PDC (flux-6) or PC (flux-7), with a relative PDC:PC ratio of 8:1. Around 44% of the PEP–pyruvate flux was diverted to the synthesis and output of lactate towards the cell culture medium (flux-8), and pyruvate cycling flux (flux 9). This finding accounted for the fact that feedback of molecules from the TCA cycle back towards the PEP–pyruvate pool was around ten-times higher than the flux of the TCA cycle towards citrate synthesis (flux-10).

### 2.2 Effect of resistin on the metabolic flux profile of L6E9

L6E9 myotubes treated with 100 nM resistin showed 13% higher flux of glucose phosphorylation (1.700 nmol · mL^–1^ · min^–1^). Fitting of the experimental data from this incubation condition using Isodyn (Table 3, second column) suggested that resistin increased the glycolytic flux, with a significant increase in the glucose phosphorylation flux, and a proportional increase of glycolytic fluxes. Resistin induced changes in the non-oxidative branch of the PPP (data not shown), but the relative flux through the oxidative branch (flux-2) was unchanged. Glycogen recycling-related fluxes (fluxes 4 and 5) remained unchanged, as indicated by the fact that the net ^13^C incorporation in glycogen was very similar to the control condition.

**Figure 2:**
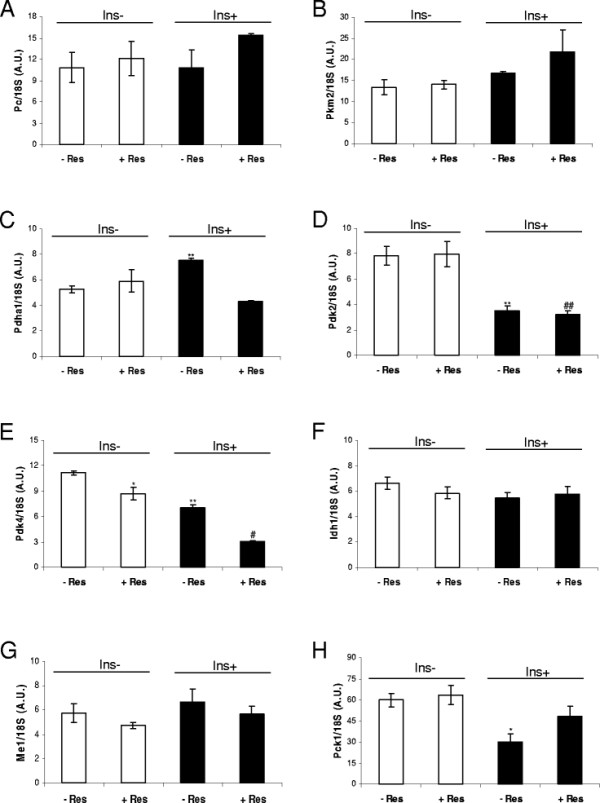
**Gene expression of enzymes related to pyruvate homeostasis.** L6E9 myotubes were treated (Res+) or not treated (Res–) with 100 nM resistin for 8 h and then incubated for 6 h with 10 mM glucose in the absence (Ins–) or presence (Ins+) of 100 nM insulin. RT-qPCR monitoring of mRNA levels of pyruvate carboxylase (*Pc*) **(A)**, pyruvate kinase muscle isozyme (*Pkm2*) **(B)**, pyruvate dehydrogenase E1 alpha 1 (*Pdha1*) **(C)**, pyruvate dehydrogenase kinase isozyme 2 (*Pdk2*) **(D)**, pyruvate dehydrogenase kinase isozyme 4 (*Pdk4*) **(E)**, cytosolic NADP-isocitrate dehydrogenase (*Idh1*) **(F)**, NADP-dependent malic enzyme (*Me1*) **(G)** and cytosolic phosphoenolpyruvate carboxykinase 1 (*Pck1*) **(H)** are shown. Results from three experiments are expressed as the mean ± S.E.M. (*) indicates p < 0.05 and (**) p < 0.01 compared with the condition of neither resistin nor insulin. (#) indicates p < 0.05 and (##) p < 0.01 compared with L6E9 myotubes treated only with resistin.

Moreover, resistin increased lactate production (Table 1), which translated to an increase in the lactate dehydrogenase flux (flux-8), though it was not significant. The most significant metabolic change after Isodyn fitting was observed in the PDC-related flux (flux-6), which was increased significantly (99% confidence). If we consider the relative ratios of the two main pyruvate incorporation fluxes (PDC and PC, fluxes 6 and 7, respectively), there was an increase of the PDC prevalence to 20:1. This finding was also supported by the level of pyruvate dehydrogenase kinase isozyme 4 (*Pdk4*) mRNA (Figure 2E), and was observed as a trend in the level of pyruvate dehydrogenase *Pdha1* mRNA (Figure 2C). Despite the large change in this ratio, the effect on fluxes in the TCA cycle was almost negligible even though there was an increase in the acetyl-CoA output flux (flux-14). The pyruvate cycling flux was unchanged by resistin treatment alone, which was supported by the level of *Pepck* mRNA (Figure 2H).

### 2.3 Effect of resistin on the metabolic response of insulin in L6E9 myotubes

Having investigated the alterations caused by resistin on the metabolic profile of L6E9 myotubes, we examined its effect on the metabolic response of myotubes to insulin.

First we ascertained if resistin affected stimulation of insulin-induced glucose transport. Thus, induction of 2-deoxy-D-[2,6-^3^H]-glucose (2-DG) transport was assayed 30 min after incubation with 100 nM insulin in L6E9 myotubes treated or not treated with 100 nM resistin. Insulin induced an increase in 2-DG transport in L6E9 myotubes of 79.27 ± 0.04%. However, it induced an increase in glucose transport in cells that had been pre-treated with 100 nM resistin for 8 h of 48.00 ± 0.05%. Subsequently, we analysed changes in the metabolic flux profile induced by insulin in L6E9 myotubes treated or not treated with 100 nM resistin.

In the absence of resistin, insulin stimulated an increase in glucose consumption and lactate production in L6E9 myotubes. There were no changes in glycogen content, but a 150% increase in intracellular G6P content (Table 1). Fitting of experimental data using Isodyn (Table 3, third column) revealed that the flux of lactate production (flux-8) was 48% higher than that in cells not treated with insulin. Insulin also increased the flux of glucose phosphorylation (determined to be 2.300 nmol · mL^–1^ · min^–1^) and glycolytic fluxes (fluxes 1 and 3) by ≈ 59%, and induced a 25% increase in the oxidative PPP flux (flux-2). Moreover, fluxes related to glycogen metabolism (fluxes 4 and 5) did not change significantly after insulin treatment (albeit the median values were apparently higher than in the control condition). Insulin increased the flux through PDC (flux-6) by around 1.5-fold, but did not affect the remainder of TCA cycle-related fluxes significantly, as confirmed by the reduction in levels of *Pdk4* mRNA and the increasing tendency of levels of *Pdha1* mRNA (Figure 2E and C, respectively). The relative ratio of PDC:PC fluxes increased from 8:1 to 13:1 after insulin treatment. Insulin also led to a 59% increase in the acetyl-CoA output fluxes.

In the presence of resistin, insulin increased the flux of glucose phosphorylation by 16% more than when cells were treated with resistin alone. Glycolysis fluxes did not increase significantly compared with the resistin condition, but the lactate dehydrogenase flux increased by 45% (flux-8). The increase induced by resistin treatment in the oxidative PPP pathway was not significantly altered if cells were also treated with insulin. Similarly, the GS and GP fluxes did not change significantly after resistin treatment.

Interestingly, there was no change in the PDC flux (flux-6) or the acetyl-CoA production flux (flux-14), and no change was observed in the remainder of TCA cycle-related fluxes. If the ratio between PDC and PC fluxes is taken into account, it shifted from 20:1 towards 70:1 after resistin was combined with insulin. The final metabolic state of insulin-stimulated L6E9 did not match the one of cells treated with resistin. Some fluxes were equal in resistin-treated and -untreated cells: oxidative PPP flux, GS flux, GP flux, and TCA cycle-related fluxes. Most of the quantified fluxes were lower in resistin, but significant changes were found only in the lactate dehydrogenase flux, and the PDC:PC ratio.

### 2.4 Effect of resistin on gene expression of the enzymes involved in the PDC, PC and pyruvate cycling fluxes in L6E9

To investigate further if the observed effects in metabolic-flux distribution were prompted by a change in the gene expression of any of the enzymes involved in their activities, we analysed the gene expression of several enzymes involved in pyruvate homeostasis at the end of the four incubation conditions tested.

Neither insulin, nor resistin, nor combination of the two hormones had a significant effect on levels of pyruvate carboxylase (*Pc*) mRNA or pyruvate kinase muscle isozyme (*Pkm2*) mRNA (Figure 2A and B). However, insulin (but not resistin) increased levels of pyruvate dehydrogenase E1 (*Pdha1*) and decreased pyruvate dehydrogenase kinase isozyme 2 (*Pdk2*) mRNA (Figure 2C and D). Presence of resistin did not alter the decrease in levels of *Pdk2* mRNA caused by insulin, but prevented the increase in levels of *Pdha1* mRNA. mRNA expression of pyruvate dehydrogenase kinase isozyme 4 (*Pdk4*) decreased after treatment with both hormones (Figure 2E), with an additive effect if both were combined. These results suggest that resistin prevents the insulin-induced increase in *Pdha1* expression, and does not prevent the increase in PDC activity caused by a reduction of expression of *Pdk2* and *Pdk4* genes.

Moreover, mRNA levels of cytosolic NADP-isocitrate dehydrogenase (*Idh1*) and NADP-dependent malic enzyme (*Me1*), both causes of pyruvate cycling increasing NADPH production, were not affected by any of the hormones tested under these conditions (Figure 2F and G). However, insulin significantly decreased (by 50%) levels of *Pck1* mRNA in the absence of resistin, but not in the presence of resistin (Figure 2H). These results suggest that resistin prevents inhibition of expression of the *Pck1* gene. Hence, cytosolic flux in PEPCK may be active in the presence of resistin independent of the presence of insulin.

## 3 Discussion

After the discovery of several adipocyte-secreted molecules (including lipid metabolites and adipokines), adipocytes were recognized as part of endocrine tissue [9],[11],[37]. These molecules enable adipocytes to communicate with other tissues and organs, and to regulate: the metabolism of lipids and glucose; energy balance; insulin action; cell proliferation. Resistin was identified as an adipokine, and it was suggested that it might link obesity and IR [12],[13]. However, the role of resistin in the pathophysiology of IR in humans and animals, and how it acts in muscle, liver and fat, is controversial [15],[16].

Here, we investigated the effect of resistin on basal and insulin-stimulated glucose metabolism in L6E9 rat myotubes. We used tracer-based metabolomics and our *in-house* Isodyn software to analyse quantitatively metabolic flux distribution in this cell line under different incubation conditions. The response to insulin and other hormone stimuli in L6E9 cells is similar to that observed in skeletal muscle *in vivo*[38]. Hence, L6E9 cells are considered to be suitable models for analyses of the effect of resistin on glucose metabolism and on the metabolic response of muscle cells to insulin.

Analyses with Isodyn data provided an overview of the central carbon metabolism of L6E9 skeletal muscle cells. Analyses were undertaken assuming a metabolic steady state (though not assuming an isotopic steady state), and rapid mixing of isotopic isomers of the same metabolite throughout different intracellular compartments. Incubation with a large excess of glucose ensured the required glucose uptake throughout the incubation. The other conditions of incubation did not change, so the assumption of a metabolic steady state seemed reasonable. Rapid mixing of isotopic isomers of the same species throughout the cell is accepted for analyses of data of stable isotopes [39]. Fitting of experimental data in the framework of these assumptions suggested a set of metabolic fluxes underlying the measured distributions of isotopic isomers.

### 3.1 The effect of insulin on L6E9 myotubes increases glycolysis, PPP, and incorporation of pyruvate to the TCA cycle through PDC and fatty-acid synthesis

Our results suggested that L6E9 myotubes were highly glycolytic, with active synthesis and degradation of glycogen, and showed TCA-cycle activity and acetyl-CoA output. Oxidative PPP was weakly active in this cell type. Furthermore, use of the same tools revealed that insulin activated the uptake and phosphorylation of glucose, glycolysis and lactate production, the oxidative branch of the PPP, pyruvate incorporation towards the TCA cycle and increased acetyl-CoA output (which is used for fatty-acid synthesis). We observed that insulin altered the ratio between PDC and PC fluxes by increasing the PDC flux through an increase of levels of *Pdha1* and decrease of *Pdk2* and *Pdk4* mRNA. The effects of insulin on the metabolic flux distribution of L6E9 myotubes reported in the present study correlate with the known activation of PDC by profound suppression of *Pdk4* expression in skeletal muscle [40], inhibition of expression of the *Pck1* gene, and the increase in glucose uptake and lactate production observed using other approaches [38],[41].

### 3.2 The effect of resistin on L6E9 myotubes increases glycolysis and fatty-acid synthesis and alters the ratio between PDC and PC, tilting it towards direct incorporation of pyruvate through PDC

Our results revealed that, in the absence of insulin, resistin activated glucose phosphorylation and the glycolytic pathway. Also, resistin did not affect the glycogen content in skeletal muscle. Even though enrichment of the glycogen label (reflected in the m2 values of the glycogen molecule) was not significantly different to the control condition, the tendency was for glycogen-related fluxes to be lower, a result that was in accordance with other works [22]. Furthermore, our approach showed that, even though many of the TCA-cycle fluxes remained unchanged, the ratio between the PDC and PC fluxes was significantly altered, effectively increasing the incorporation of pyruvate through PDC into mitochondria (as suggested by the reduction observed in levels of *Pdk4* mRNA). PC activity is finely regulated by mechanisms such as allosteric activation by acetyl carnitine, which was not tested in this study. The acetyl-CoA output for fatty-acid synthesis was also increased after resistin treatment, which would explain the higher flux towards the TCA cycle.

### 3.3 The effect of resistin impairs normal insulin metabolic actions

We showed that resistin affects the normal metabolic response of L6E9 myotubes to insulin. Thus, the increase in glucose uptake and glycolytic flux induced by insulin was lower than that observed in resistin-treated myotubes. These results are in accordance with those published by other authors for L6 muscle cells [19],[21],[22]. Resistin treatment, however, did not affect the insulin-stimulated content and recycling of glycogen.

Moreover, in resistin-treated cells, insulin did not increase the PDC flux. After resistin treatment, levels of *Pdk2* mRNA and *Pdk4* mRNA were lower, accompanied with a reduction in levels of *Pdha1* mRNA. The reduction in expression of the main enzyme and key regulators of its activity resulted in a null net effect on the final flux. The ratio between the PDC and PC fluxes was also altered, showing reduction in the PC anaplerotic entrance into the cycle.

Fluxes through the TCA cycle and acetyl-CoA output fluxes were not increased, but instead maintained or reduced, by insulin in resistin-treated cells. This lack of metabolic activation in response to insulin can be explained by the slight decrease observed in glucose uptake and the increase of lactate dehydrogenase flux directed to lactate production, which impaired the increase in the levels of glycolytic intermediates that could be metabolized through other metabolic pathways. Our results provide mechanistic explanations for the observations of Palanivel and co-workers [22], who reported that the insulin-stimulated oxidation of glucose *via* the TCA cycle was reduced by resistin, and that this reduction would compromise oxidative respiration in the cell.

## 4 Conclusions

Several studies have identified positive correlations between resistin levels and IR *in vivo*[24] and *in vitro*[19]-[22]. Overall, our results showed that resistin significantly alters glucose metabolism in L6E9 myotubes and their response to insulin. Of the whole set of metabolic changes induced by resistin in L6E9 myotubes, the imbalance in PDC and PC fluxes as well as the higher acetyl-CoA output flux for fatty-acid synthesis and other metabolic processes should be emphasized. Resistin impairs the normal metabolism of insulin, and the metabolism is directed to try to maintain the imbalance between the PDC and PC fluxes. In this way, L6E9 reduces TCA-cycle fluxes and acetyl-CoA output fluxes, which leads to an important metabolic imbalance in the central carbon metabolic pathways of L6E9 myotubes.

It has been reported that IR in skeletal muscle, liver and adipose tissue is usually accompanied by glucose intolerance and hypertriglyceridemia [24]. In this way, our results showed the importance of the balance of PDC and PC in glucose metabolism.

Our results support the idea that metabolic disorders such as T2DM are caused by complex multi-molecular interactions that cannot be explained readily by an alteration in expression of a single gene or gene product, or even alteration of a single enzymatic cascade. The robustness of results obtained on the effects of insulin on L6E9 metabolism found by combining tracer-based metabolomic data and flux analyses using Isodyn showed that this approach is a suitable tool to study the effects of hormones on the central carbon metabolism network in myocytes.

## 5 Methods

### 5.1 Cells and reagents

Myoblastic cell line L6E9 was kindly provided by Dr. Nadal-Ginard (Harvard University, Boston, MD, USA). Rat recombinant resistin was produced in a HEK293 cell line. The purity of resistin was controlled by sodium dodecyl sulfate–polyacrylamide gel electrophoresis (SDS-PAGE) and estimated to be >99%. Biological activity was validated as described [42]. Resistin was tested for endotoxin using a ToxinSensor Chromogenic LAL Endotoxin Assay kit (bioNova científica, Madrid, Spain). This assay indicated low levels of endotoxins (0.0015 EU/mL) at the final concentration used, which is below current USA Food and Drug Administion (FDA) limits that require eluates from medical devices to be <0.5 EU/mL. [1,2-^13^C_2_]-D-glucose and [U-^13^C-^2^H_7_]-D-glucose were purchased from Isotec (Miamisburg OH, USA) and 2-deoxy-D-[2,6-^3^H]-glucose (2-DG) from GE Healthcare (Barcelona, Spain).

### 5.2 Conditions for cell culture and incubation

Myoblastic cell line L6E9 was grown as described [38]. For biochemical and mass isotopomer assays, L6E9 myoblasts were seeded at 10^4^ cells/cm^2^ in 60-mm diameter dishes with 4 mL Dulbecco’s modified Eagle medium (DMEM) without glucose or glutamine (Biological Industries, Kibbutz Beit Haemek, Israel) supplemented with 10% heat-inactivated foetal bovine serum (FBS) (Life Technologies, Madrid, Spain), 10 mM glucose, 4 mM asparagine and 5% penicillin/streptomycin (Sigma–Aldrich, Saint Louis MO, USA). Cell differentiation was induced by lowering FBS to a final concentration of 2% when myoblasts were pre-confluent [38].

Cells at day-4 of differentiation were washed four times in phosphate-buffered saline (PBS) and pre-treated or not with 100 nM resistin dissolved in DMEM without FBS and supplemented with 10 mM glucose, 4 mM asparagine, 2% HEPES (Sigma–Aldrich) and 1% penicillin/streptomycin. After 8 h, the medium was replaced by another with the same composition, but now with 10 mM glucose 50%-enriched in [1,2-^13^C_2_]-glucose in the presence or absence of 100 nM insulin (Sigma–Aldrich). At the end of 6-h incubation, media were collected and dishes washed twice with PBS before freezing in liquid nitrogen. Incubation media and dishes were stored at –80°C until biochemical and mass isotopomer analyses.

### 5.3 Glucose transport assay

To carry out the glucose transport assay, 10^5^ L6E9 cells/dish were cultured on six-well plates. At day-4 of differentiation, cells were pre-treated or not with 100 nM resistin for 8 h, as described above. Next, uptake of 100 μCi 2-DG by myotubes incubated with or without 100 nM insulin for 30 min was measured as described [38].

### 5.4 Biochemical determinations of glucose, lactate and G6P

Concentrations of glucose and lactate in incubation media at the beginning and end of 6-h incubations were measured as described previously by spectrophotometric methods coupled to a Cobas Mira Plus Chemical Analyzer (HORIBA ABX, Montpellier, France). Intracellular G6P content was determined by enzymatic means as described [43].

### 5.5 Mass isotopomer analyses of lactate, medium and glycogen glucose, and RNA ribose, and determination of glycogen content by GC/MS

Mass spectral data were obtained on a QP2010 Shimadzu Mass Selective Detector connected to a GC-2010 Gas Chromatograph (Shimadzu, Kyoto, Japan) using helium as the carrier gas and isobutane 0.0016 Pa as reagent gas in chemical ionization analyses. The settings were: GC inlet, 250°C for glucose, ribose and deoxyribose, and 200°C for lactate; transfer line, 250°C; MS source, 200°C. A Varian VF-5 Capillary Column (30-m length, 250-μm diameter, 0.25-μm film thickness) was used to analyze all compounds.

Glucose from the frozen media was purified using a tandem set of Dowex-1X8/Dowex-50WX8 (Sigma–Aldrich) ion-exchange columns. The glucose from media was converted to its glucose aldonitrile pentaacetate derivative as described [43]. Ion clusters around *m/z* 328 (carbons 1–6 of glucose, chemical ionization) were monitored.

Lactate from the media was extracted with ethyl acetate after acidification with hydrochloric acid (HCl) and derivatized to its propylamide-heptafluorobutyric form. Clusters around *m/z* 328 (carbons 1–3 of lactate, chemical ionization) were monitored [43].

RNA ribose was isolated by acid hydrolysis of cellular RNA after Trizol purification of cell extracts. Ribose isolated from RNA was derivatized to its aldonitrile-acetate form using hydroxylamine in pyridine and acetic anhydride. We monitored ion clusters around *m/z* 256 (carbons 1–5 of ribose, chemical ionization) to find the molar enrichment and positional distribution of ^13^C labels in ribose as described [44].

Glycogen in frozen cell monolayers from different analytical conditions was extracted and quantified as described [44]. Extraction was done by direct digestion of sonicated extracts with amyloglucosidase. Mass isotopomer analyses of glycogen glucose was undertaken as described above for medium glucose. Measurement of glycogen content was carried out using the isotopomer [U-^13^C-^2^H_7_]-glucose as recovery standard and internal standard quantification procedures. The ion cluster for the [U-^13^C-^2^H_7_]-glucose of the glucose aldonitrile-pentaacetate derivative was monitored from *m/z* 339 to *m/z* 341.

Spectral data were corrected using regression analysis to extract natural ^13^C enrichment from results [45]. Measurement of ^13^C label distribution determined the different relative distribution percentages of the mass isotopomers, m0 (without any ^13^C labels), m1 (with one ^13^C), m2 (with two ^13^C), etc., which were reported as molar fractions.

### 5.6 Analyses and modelling of data using Isodyn

Transfer of ^13^C from [1,2-^13^C_2_]-glucose medium into intracellular metabolites was simulated by Isodyn, a software program written in C++ and designed for analyses of data from stable isotopic tracers [26],[33],[34]. Latest modifications of this software have been described in detail [35],[36].

This software automatically constructs and solves the large system of ordinary differential equations describing the evolution of isotopomer concentrations of metabolites produced in glycolysis, TCA cycle, and PPP (Figure 1). All the reactions of the non-oxidative branch catalyzed by transketolase and transaldolase are accounted for, assuming pentose-5-phosphates to be a single pool. The reactions of the oxidative branch are passed to one reaction leading from G6P to pentose-phosphates. At this initial moment, all metabolites (except added labeled substrate) are assumed to be non-labeled, and the latter is set in accordance with the known isotopomer composition of the used labeled substrate. Initial total concentrations of intracellular metabolites are calculated as functions of model parameters assuming a steady state at the initial moment. Functions designed especially for each type of reaction (e.g., carboxylation, decarboxylation, transketolase-type) simulate transformation of the carbon skeleton (specific transitions of labeled carbons), and the production and consumption rate for each isotopomer of the considered system. These transformations redistribute ^13^C isotopes in all metabolites. Individual rates are computed for each isotopomer, and these rates determine the values of the derivatives for the isotopomers. This system is solved using a method of numerical integration that can be chosen arbitrarily (Runge-Kutta, BDF, Dassl). Starting from the initial values corresponding to the experimental conditions of incubation, Isodyn simulates a real-time course of label propagation. Experimental and computed data for corresponding time points are compared. In this way, reaching an isotopic steady state is not required.

### 5.7 RNA extraction and real-time RT-qPCR gene-expression analyses

Total RNA was isolated from L6E9 cells using an RNeasy Mini kit and a QIAcube Automat (Qiagen, Venlo, the Netherlands). RNA integrity was checked by capillary electrophoresis (Experion; Bio-Rad, Hercules, CA, USA). Concentrations were checked with Ribogreen (Molecular Probes, Eugene, OR, USA) and a Victor-X5 Multilabel Reader (PerkinElmer, Waltham, MA, USA). Labelled cDNA was synthesized from 600 ng of total RNA using SuperScript-II, reverse transcriptase and random hexamers (Life Technologies, Madrid, Spain).

Oligonucleotide sequences for the reverse transcriptase reaction, synthesized by Eurogentec (Angers, France), were: pyruvate carboxylase (PC), *Pc*, rPC-F, TTCCGTGTCCGAGGTGTAAAG, and rPC-R, CGCTAGGAACTGCTGGTTGTT; pyruvate kinase muscle isozyme, *Pkm2*, rPKm-784F, GAGCAGGACGTGGACATGGT, and rPKm-864R, CTCTCCCAGGACCTTCCTAACC; pyruvate dehydrogenase E1 alpha 1, *Pdha1*, rNm_001004072.2F, TTTGTCTTCTGTGCTGGGAGACTG and rNm_001004072.2R, GTAGATGGGTGGCTTCAAGTTTGC; pyruvate dehydrogenase kinase isozyme 2, *Pdk2*, rNm_030872.1F, CATGGCTAAGCTCCTGTGTGAC, and rNm_030872.1R, GGACGTAGACCATGTGAATGGG; pyruvate dehydrogenase kinase isozyme 4, *Pdk4*, rNm_053551.1F, TGCTCATGAACCAGCACATCCTC and rNm_053551.1R, TCCCAATGTGGCTTGGGTTTCC; cytosolic NADP-isocitrate dehydrogenase, *Idh1*, rNm_031510.1F, AGACGTCCACCAATCCCATTGC, and rNm_031510.1R, TCAAGCTTTGCTCTGTGGGCTAAC; NADP-dependent malic enzyme, *Me1*, rNm_012600.2F, TGCAAGACCATGGTTCCCAGAC, and rNm_012600.2R, AAGCTAAGCCCAGGGACATTAGGG; cytosolic phosphoenolpyruvate carboxykinase (PEPCK) 1, *Pck1*, rPEPCK1-2228F, TGGTTCCACTTCGAGGTCACT, and rPEPCK1-2310R, CAAGTATGTTTTCTGTGCACTTTAGCT.

Real-time PCR (qPCR) was conducted with a Sybrgreen Master Mix Reagent using Sureprime Core kit (MP Biomedical, III Kirch, France) in an iCycler (Bio-Rad, Marnes-la-Coquette, France) apparatus. The standard curve method was used for relative quantification of PCR products, and gene expressions normalized to 18S RNA quantification, which has been found to be a reliable internal control gene in our hands and by other scholars [46]-[48].

### 5.8 Statistical analyses

Experiments relating to glucose transport were carried out using three cultures each time for each treatment regimen and then repeated thrice. Metabolic characterization assays were carried out in triplicate and repeated twice. RNAs for RT-qPCR assays were isolated from three independent extracts. Statistical analyses of glucose transport as well as biochemical and metabolite mass isotopomer distributions were calculated by two-way ANOVA between groups. Data for real-time PCR were analyzed by the one-way ANOVA between groups. Confidence intervals at a confidence level of 95% or 99% (p < 0.05 and p < 0.01, respectively) were taken as indicating significant differences in each parameter analyzed. Differences from the mean were considered significant for p < 0.05.

In the case of the metabolic fluxes estimated using Isodyn, the difference between calculated and experimental data (χ^2^) was minimized using a simulated annealing algorithm. The goodness of best fit (corresponding to the minimal deviation of calculated isotopomer fractions from the experimental ones) was checked based on the value of χ^2^ and number of degrees of freedom [36]. In the case that, according to this checking, the fit was deemed acceptable, the metabolic fluxes corresponding to the best fit were accepted as consistent with the measured isotopomer distribution. The confidence intervals for the fluxes were determined as the upper and lower limits of fluxes that produced χ^2^ that was less than the fixed threshold [36].

## Abbreviations

2-DG: 2-deoxy-D-[2,6-^3^H]-glucose

G6P: Glucose-6-phosphate

GP: Glycogen phosphorylase

GS: Glycogen synthase

IR: Insulin resistance

PC: Pyruvate carboxylase

PDC: Pyruvate dehydrogenase complex

PEP: Phosphoenolpyruvate

PEPCK: Phosphoenolpyruvate carboxykinase

PPP: Pentose phosphate pathway

T2DM: Type-2 diabetes mellitus

TCA: Tricarboxylic acid

## Competing interests

The authors declare that they have no competing interests.

## Authors’ contributions

SG, SM, FS, AZ, PR and MC conceived and designed the research. SG, SM, AM, RH, FS, AT and YD undertook the experiments. SG, SM, VS, AM, RH, FS, AT, YD and MC analysed data. SG, SM, AM, VS, JC, AZ, PR, and MC interpreted the results of the experiments. SG, SM, JC, AM, RH, FS, AT, YD and PR prepared the figures. SG, SM, AM, VS, PR and MC drafted the manuscript. SG, SM, AM, VS, JC, AZ, PR and MC edited and revised the manuscript. All authors read and approved the final manuscript.
